# Functionality of Reactive Oxygen Species (ROS) in Plants: Toxicity and Control in *Poaceae* Crops Exposed to Abiotic Stress

**DOI:** 10.3390/plants13152071

**Published:** 2024-07-26

**Authors:** Sanjib Kumar Panda, Divya Gupta, Mayur Patel, Christell Van Der Vyver, Hiroyuki Koyama

**Affiliations:** 1 Department of Biochemistry, Central University of Rajasthan, Ajmer 305817, India; sanjib.panda@curaj.ac.in (S.K.P.); gupta.divya.biotech20@gmail.com (D.G.); mayurkpatel26499@gmail.com (M.P.); 2 Institute of Plant Biotechnology, Stellenbosch University, Private Bag X1, Stellenbosch 7601, South Africa; cvdv@sun.ac.za; 3 Faculty of Applied Biology, Gifu University, Gifu 501-1193, Japan

**Keywords:** ROS, abiotic stress, antioxidant, signaling, drought

## Abstract

Agriculture and changing environmental conditions are closely related, as weather changes could adversely affect living organisms or regions of crop cultivation. Changing environmental conditions trigger different abiotic stresses, which ultimately cause the accumulation of reactive oxygen species (ROS) in plants. Common ROS production sites are the chloroplast, endoplasmic reticulum, plasma membrane, mitochondria, peroxisomes, etc. The imbalance in ROS production and ROS detoxification in plant cells leads to oxidative damage to biomolecules such as lipids, nucleic acids, and proteins. At low concentrations, ROS initiates signaling events related to development and adaptations to abiotic stress in plants by inducing signal transduction pathways. In plants, a stress signal is perceived by various receptors that induce a signal transduction pathway that activates numerous signaling networks, which disrupt gene expression, impair the diversity of kinase/phosphatase signaling cascades that manage the stress response in the plant, and result in changes in physiological responses under various stresses. ROS production also regulates ABA-dependent and ABA-independent pathways to mitigate drought stress. This review focuses on the common subcellular location of manufacturing, complex signaling mechanisms, and networks of ROS, with an emphasis on cellular effects and enzymatic and non-enzymatic antioxidant scavenging mechanisms of ROS in *Poaceae* crops against drought stress and how the manipulation of ROS regulates stress tolerance in plants. Understanding ROS systems in plants could help to create innovative strategies to evolve paths of cell protection against the negative effects of excessive ROS in attempts to improve crop productivity in adverse environments.

## 1. Understanding Reactive Oxygen Species: An Overview

Plants face a variety of biotic and abiotic stresses, which result in decreased yields and threaten the sustainable cultivation of commercially significant crops. On an annual basis, crop production needs to overcome a lack of water, excessive global temperatures, increased soil salinity, herbicidal incursions, and pest and pathogen attacks. Especially frequent and severe droughts due to abnormal weather conditions are considered the main threat to sustainable global crop production [1]. Plants are immobile and cannot migrate to escape oxidative stress caused by water limitations. However, the capacity of plants to sense stress incidences and behave accordingly by activating complex signaling mechanisms helps plants adapt, accomplish, and modify their level of tolerance towards stress [2,3]. Under abiotic stresses such as drought, salt, heat, cold, heavy metal, etc., ROS causes both metabolic activity disruption and activation of significant regulatory mechanisms by inducing secondary messengers like Ca^+2^.

Under drought stress, abscisic acid root-to-leaf signaling takes place to trigger the stomatal closure [4], which limits carbon dioxide fixation, which results in reduced net photosynthesis and also affects ROS accumulation in plant cells [5]. In wheat, increased electron leakage was studied through the Mehler reaction during photosynthesis under drought stress [6]. Similarly, in sunflowers, electron leakage was observed in the thylakoid membrane under drought stress [7]. As drought induces oxidative stress, cultivated crops have to deal with an oxidative load by activating ROS scavenging mechanisms, which involve enzymatic and non-enzymatic antioxidant responses [8]. Under water deficit stress, the equibalance is disorganized between ROS accumulation and the activity of antioxidant molecules [9]. Over the years, plants have adapted to react to the mischievous action of ROS, and these antioxidation mechanisms can potentially be enhanced in crop species to counter the negative effects of oxidative stress.

**Figure 1:**
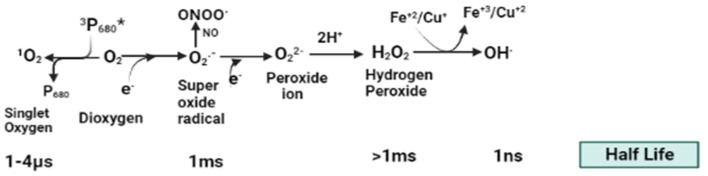
Different forms of reactive oxygen species (ROS) and their reactivity. This figure illustrates various forms of ROS, highlighting their half-lives and corresponding reactivities. Here P_680_* is an electron donor to an acceptor.

Reactive oxygen species are chemical molecules and free radicals derived from molecular oxygen found in all aerobic life [10]. It includes O_2_^•−^ (superoxide radical anion), OH^•^ (hydroxyl radical), H_2_O_2_ (hydrogen peroxides), ^1^O_2_ (singlet oxygen), etc. (Figure 1). In crops, a stress-indoctrinated signal transduction pathway is usually activated by a stress signal, which is perceived by receptors such as receptor kinases, histidine kinases, tyrosine kinases, and G-protein-coupled receptors (GPCR), along with receptors of ROS and other stress-induced metabolites and molecules [11]. Dealing with oxidative stresses by these receptors operates numerous complex signaling networks in plants, disrupts the expression of genes, and impairs the diversity of kinase/phosphatase signaling cascades that manage the action of stress in plants [12]. Moreover, the action between ROS and phytohormones could act as a boost curve to synchronize gene expression and change physiological responses under various stresses [13]. Respiratory burst oxidase homolog-like (RBOH) proteins are a key driving force that intricately regulates several signaling pathways in plants in a ROS-dependent manner [9]. These RBOH proteins are located in the plasma membrane as NADPH oxidases. Under stress conditions, these RBOH proteins are controlled by the phosphorylation or binding of calcium (Ca^+2^) ions or other molecules localized in the cytosol, which form superoxide (O_2_^•−^radicals in the apoplast. This superoxide is catalyzed into H_2_O_2_ by the superoxide dismutase enzyme. High concentrations of ROS led to oxidative damage to lipids, nucleic acids, and proteins, which are essential for enzyme activity [14]. ROS is also a secondary messenger involved in signaling cellular functions like cell proliferation, necrosis, and apoptosis [15]. Changing environmental circumstances increase the oxidative load, which adversely affects plant development and physiological feedback [16].

This review presents a classical account of the diverse nature of ROS in various plant stress signals. It also discusses the interaction of ROS with various phytohormones under abiotic stress conditions, such as drought, by regulating systemic and acquired plant responses to adapt to adverse environmental conditions, with a specific focus on *Poaceae* crop species. Understanding the involvement of ROS in plants’ stress response mechanisms and complex signaling networks will continuously innovate new strategies, like constructing pathways of cell safeguarding against the negative action of ROS accumulation, in crops to rectify plant yield under the harshness of environmental stress and the increasing global requirement for food production.

## 2. How Are Reactive Oxygen Species (ROS) Generated?

Oxygen (O_2_), the source molecule of all ROS, is stable and not especially reactive in plants. However, it can be altered into high-energy ROS in different plant organelles by assorted processes that affect plant metabolism. The baseline of ROS production is a necessary step to synchronize normal plant growth and development. In general, low ROS levels are crucial for biological processes such as cellular proliferation and differentiation [17,18]. In addition, as an important signaling molecule, ROS also regulates the plant’s response to stress. This dual role of ROS is conditional on the distinct levels of reactivity, sites of production, and capability to cross biological membranes [19].

Numerous abiotic stresses lead to the assembly of ROS, which causes cytotoxicity in diverse cellular compartments. During light stress, there is the production of O_2_^•−^, ^1^O_2_, and H_2_O_2_ in the chloroplast primarily, and in C3 plants, H_2_O_2_ starts to accumulate in the peroxisome [20,21]. ROS profiles differ in C3 and C4 plants primarily because of reduced photorespiration in the latter [22]. C4 plants, such as sugarcane, sorghum, and maize, concentrate CO_2_ around ribulose-1,5-bisphosphate carboxylase-oxygenase (RuBisCO) in bundle sheath cells. RuBisCO is the enzyme responsible for the fixation of CO_2_ during photosynthesis. Plants that utilize C4 photosynthesis are known to have evolved at lower atmospheric CO_2_ and higher O_2_ levels [23]. Abiotic stressors cause photorespiration by decreasing stomatal conductivity, leading to a decrease in intercellular CO_2_ levels, which also influences RuBisCO kinetics [23]. This implies that C4 plants might potentially be more robust than C3 plants under photorespiration-inducing abiotic stresses. Under drought conditions, an upsurge in ROS production has been observed due to limited CO_2_ availability due to stomatal closure in C3 and C4 plants such as *Arabidopsis* and crops like sugarcane, maize, sorghum, and rice [24,25,26,27,28,29,30,31]. Elevated levels of ROS were also observed in plants exposed to heat stress, resulting in heightened production of O_2_^•−^ and H_2_O_2_ with electron transport chain (ETC) disruption in mitochondria [32]. With the current technological advancements, ROS transportation, accumulation, and modulation of retrograde and anterograde signaling between different cellular compartments and nuclei have been identified and highlighted under specific stress conditions [33,34,35,36]. To understand regulatory mechanisms, from the accumulation of ROS to defense responses adapted at the cellular level, ROS sensing, scavenging, and transportation need to be analyzed.

## 3. Sensing Reactive Oxygen Species: Key Biological Processes

**Figure 2:**
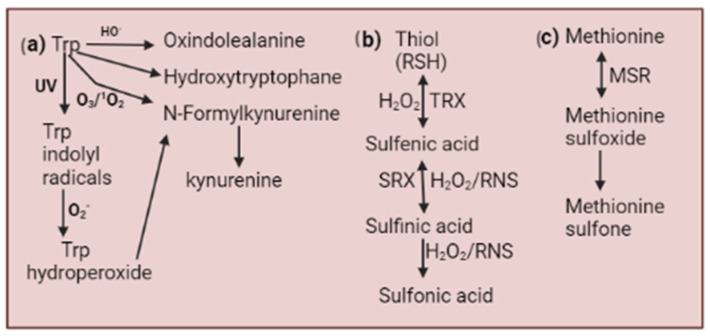
Oxidative post-translational modifications of (**a**) tryptophan (Trp), (**b**) cysteine, and (**c**) methionine. This figure depicts the oxidative modifications of tryptophan (Trp), cysteine, and methionine and their associated repair mechanisms. TRX: thioredoxin; SRX: sulfiredoxin; MSR: methionine sulfoxide reductase, UV: ultraviolet; RNS: Reactive Nitrogen Species; RHS: Alkyl thiol.

Alterations in ROS levels in stressful environments can be sensed through their oxidizing properties. An enhanced level of ROS can modify the structural and functional properties of proteins through oxidative post-transcriptional modification (PTM), which can affect several intriguing signaling pathways [37,38,39,40,41]. The primary mark of ROS is the thiol group (sulfur atom) in cysteine and methionine. ROS attack thiol groups of cysteine and form a highly reactive intermediate, sulfenic acid, which facilitates the irreversible oxidation of sulfinic and sulfonic acids, but sulfinic acid can be reversible in some cases in the presence of sulfiredoxin (Figure 2) [42,43,44]. Likewise, the thiol group of methionine is oxidized to methionine sulfoxides, which are further converted to methionine sulfone. Similarly, tryptophan is also involved in protein–protein interactions [45,46]. In the presence of ^1^O_2_ or ozone, Trp is oxidized to kynurenine and N-formylkynurenine (NFK). Under UV conditions, tryptophan produces tryptophan indolyl radicals that are further converted to kynurenine and NFK in the presence of O_2_^•−^. Trp produces oxindole, alanine, and hydroxytryptophan in the presence of HO. radicals (Figure 2) [47,48]. Thus, ROS-mediated PTM can alter protein structure or function, which can impact signal transduction pathways. Furthermore, oxi-PTM of cysteine residue might be targeted by antioxidant enzymes like peroxidase (PRX) and glutathione peroxidases (GPX), which can be characterized as ROS sensors. There is the formation of redox relays by the interaction between sensor proteins and effector proteins, such as GPXL-like 3-ABI2. The ROS sensor is the only example of a redox relay involved in stomata closure [49]. This study suggests that GPXL3 is located on the endoplasmic reticulum (ER), its catalytic domain is directed towards the lumen area, and ABI2 is present in the cytosolic region, which makes their interaction unfavorable in plants [50]. It raises concerns about the ABI2 interaction with another specified ROS sensor or other GPXLs, or whether it can sense H_2_O_2_ directly [51]. In yeast, there is a redox relay mechanism in which GPX3 oxidizes the Yap1 transcription factor and regulates transcriptional regulation [52]. The ascorbate-glutathione (ASC-GSH), a non-enzymatic ROS scavenging pathway, acts as a first-line defense against ROS accumulation. It also acts as a ROS sensor due to a change in the GSH to GSSG ratio that led to the activation of oxi-PTM of thiol of Cys residues, RBOHs, transcription factors, etc., which are potentially mediated by S-glutathionylation [53].

## 4. Exploring the Signaling Functions of Reactive Oxygen Species (ROS)

ROS sensing and signaling are regulated by different subcellular compartments. Based on this, it can be divided into the following three levels: (i) extrinsic, which includes the cell wall and apoplast; (ii) intrinsic, signaling in the cytosol and nucleus; and (iii) organelle, mitochondria, chloroplast, and peroxisome, etc. [53].

### 4.1. Extrinsic: Cell Wall and Apoplast

**Figure 3:**
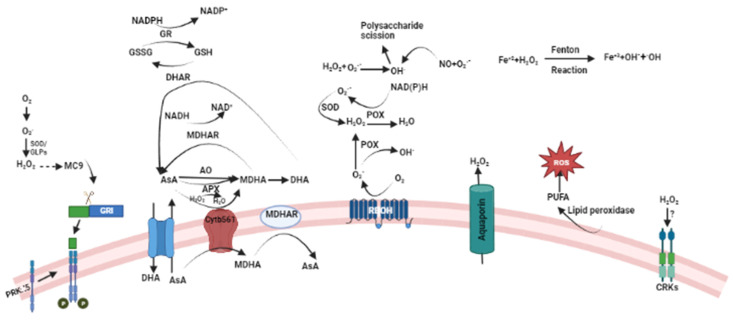
ROS production and scavenging in the apoplast region. This figure illustrates the dynamics of ROS production and scavenging within the apoplast region. It highlights the sources of ROS generation, including enzymatic activities and environmental factors, as well as the mechanisms involved in their scavenging and neutralization.CRK: cysteine-rich RLKs; MDHAR: monodehydroascorbate reductase; DHA: dehydroascorbate; GSSG: oxidized glutathione; SOD: superoxide dismutase; POX: polyamine oxidase; APX: ascorbate peroxidase; AsA: ascorbic acid; PUFA: polyunsaturated fatty acid; MC9: METACASPASE 9, GLPs: Glucagon-like peptides; P: Phosphate; PRK5: Pollen Receptor Like Kinase 5; NADPH, NADP+: Nicotinamide adenine dinucleotide phosphate; GSH: Glutathione; GR: Glutathione reductase; DHAR: Dehydroascorbate reductase; NADH, NAD+: Nicotinamide adenine dinucleotide; AO: Ascorbate oxidase; Cytb561: Cytochrome b561; MDHA: Monodehydroascorbate; RBOH: Respiratory burst oxidase homolog.

In the cell wall and apoplast, the production of ROS takes place through the action of several enzymes, which also produce several antioxidants to scavenge ROS in an enzymatic and non-enzymatic manner. The accumulation of ROS and its signaling in the apoplast results in the formation of cell wall-linked peroxidases, RBOHs, aquaporins, and polyamine oxidases [54,55,56]. Cell wall peroxidases use NADH as a substrate to promote H_2_O_2_ production, which is produced by the oxidation of malate and lactate in the presence of malate dehydrogenase and lactate dehydrogenase, respectively [57]. RBOHs, transmembrane proteins, use cytosolic NADPH to generate O_2_^•−^, which is further catalyzed by superoxide dismutase (SOD) to produce H_2_O_2_ in the apoplast [18,58,59]. Regulation of ROS is performed by the binding of calcium ion(s), phosphorylation of the cytosolic N and C terminals of RBOH, bonding of ROP-GTP, and phosphatidic acid [53]. RBOHs, as ROS signaling engines, are also influenced by nitrosylation, ubiquitination, endocytosis, glutathionylation, etc. [60,61,62,63,64,65] linked to different stresses (Figure 3). Accumulation of ROS in the apoplast also takes place via aquaporins (AQP), which are regulated by acetylation, guanidinylation, and phosphorylation with regard to ROS transportation [66,67,68,69,70]. Other than this, hydroperoxidation of poly-unsaturated fatty acids (PUFA) in the presence of lipoxygenase also contributes to ROS accumulation [71]. Sensing of ROS in the apoplast region involves different mechanisms, like oxidative PTM or the action of oxidized proteins and/or metabolites [58]. The presence of several cysteine-rich peptides in the apoplastic region could be an ROS sensor [72]. Similarly, cysteine-rich kinases (CRKs) reportedly function in the ROS accumulation process in the apoplast [73], as seen through interactions between a set of CRKs and RBOH [74]. Recently, a module of GRIM REAPER (GRI)-RLK (receptor-like kinase)-POLLEN RECEPTOR-LIKE KINASE5 (PRK) has been studied as a sensing mechanism, identified in the regulation of ozone-induced cell death (Figure 3) [75,76]. Here, METACASPASE 9 (MC9) cleaves GRI, and a 12-amino acid GRI peptide binds to PRK and regulates ROS-mediated cell death [76]. In the whole process, the importance of ROS in the activation of MC9 or its role in the interaction of peptide and PRK5 is still unclear. These ROS detoxification processes in the apoplast are regulated by enzymatic and non-enzymatic antioxidants (Table 1). Ascorbate oxidase, glutathione oxidase, and catalase were reported as enzymatic antioxidants, whereas ascorbate, glutathione, polyamines, phenolic compounds, and proline were characterized as non-enzymatic antioxidants, which regulate ROS in the apoplastic region under stress.

### 4.2. Intrinsic: Organelle Level

**Figure 4:**
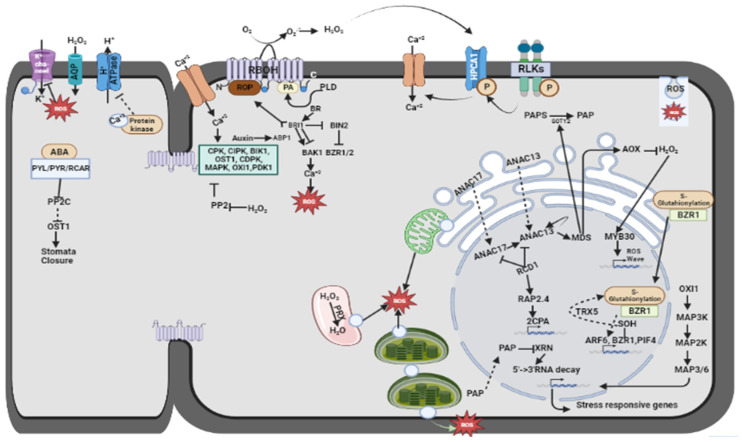
ROS production, scavenging, and transportation at an intrinsic level. This illustration emphasizes the balance between ROS production and elimination and how effective transport mechanisms contribute to cellular redox homeostasis and protection against oxidative stress. PYR/PYL/RCAR: pyrabactin resistance/pyrabactin resistance-like/regulatory component of ABA receptors; PP2C: type 2C protein phosphatase; OST1: open stomata 1; CPK: CALCIUM-DEPENDENT PROTEIN KINASE 5; CIPK: CBL-interacting protein kinases; BIK: BOTRYTIS-INDUCED KINASE 1, CDPK: calcium-dependent protein kinase; OXI1: OXIDATIVE SIGNAL-INDUCIBLE 1; PDK1: phosphoinositide-dependent kinase 1; ROP: Rho of plants; RBOH: respiratory burst oxidase homologues; BR: brassinosteroid; BIN: BRASSINOSTEROID INSENSITIVE 2; BAK: brassinosteroid (BR)-associated kinase; ABP1: ABA insensitive 1; BZR1/2: BRASSINAZOLE RESISTANT 1/2; PLD: phospholipase D; HPCA1: H_2_O_2_-INDUCED CA^2+^ INCREASES 1; RLK: receptor-like kinase; RCD1: radical-induced cell death 1; RAP2.4 RELATED TO APETALA 2.4; CPA: 2-CYS PEROXIREDOXIN A; PIF: phytochrome-interacting factor; PP2: protein phosphatase 2; XRN: exoribonuclease, ATPase: adenosine triphosphatase; PRX- Peroxidase; PAP: Purple acid phosphatase; AQP: Aquaporin; NAC: Based on three proteins no apical meristem (NAM), ATAF1–2, and cup-shaped cotyledon (CUC); SOT12: SULFOTRANSFERASE 12; MDS: Mitochondrial dysfunction stimulon; AOX: Alternative oxidase; MAPK: Mitogen-activated protein kinases; MYB: Myeloblastosis.

At the intrinsic level, changes in ROS levels are regulated by different receptors and sensors under abiotic stress, such as OSCA1 (reduced hyperosmolality-induced [Ca^2+^]i increase 1) and MSL10 (mechanosensitive channel), to detect osmotic variations. The physical propinquity of RBOHs with some of these receptors facilitates the generation of ROS at the initial stage (Figure 4) [35,36,92,93,94]. After stress perception, the initiation of responses occurs within seconds to minutes [95,96,97,98], which is coordinated by redox alteration, increased calcium ion accumulation, (de)phosphorylation, and other stress regulatory downstream signaling [53]. These activated pathways also modify hormonal regulation. Brassinosteroid (BR) binds to its receptor BRASSINOSTEROID INSENSITIVE1 (BRI1) and inactivates BRASSINOSTEROID INSENSITIVE2 (BIN2) by phosphorylation (Figure 4) [99]. Recent studies also suggested enhanced cytosolic calcium ion concentration induced by BR in a receptor-dependent manner. The guanylyl cyclase domain in BRI1 produces cGMP to regulate BR-mediated calcium ion accumulation in the cytosol [100]. BR was also reported to control PIN2 localization and induce auxin signaling. Further, both auxin and BR bind to the AUXIN BINDING PROTEIN (ABP1) and BRI1, respectively, and regulate activation of ROP-GTPase to mediate ROS production [101,102,103]. Additionally, high BR levels also regulate abscisic acid (ABA) biosynthesis under high ROS accumulation and lead to stomatal closure [104].

ROS accumulation and activation of redox sensors also modulate TFs to regulate stress-responsive gene expression. Transcriptional regulation is mediated by the following factors: (i) ROS-induced phosphorylation, calcium binding, sumoylation, etc., and (ii) redox process regulation by ROS in a direct or indirect manner [105,106,107,108,109]. Subunits of mediator complexes, which link different TFs to RNA Pol II, are also modulated by redox regulation under stress conditions. Hence, an alteration in ROS accumulation led to changes in miRNA and mRNA levels [110,111,112]. ROS also affects the translocation of transcriptional regulators such as ANAC013, ANAC017, MBF1C, HSFA1A, etc. from the cytosol or ER to the nucleus to modulate stress-responsive genes [105,106,108,113]. Under excess light conditions, enhanced ROS led to the inactivation of SAL1, which led to PAP accumulation in chloroplasts and the negative regulation of gene expression in the nucleus (Figure 4) [38,114]. Similarly, the level of PAP in the mitochondria is due to a perturbed ETC complex and stimulates ANAC013 and ANAC017 translocation to the nucleus, which are natively regulated by RCD1 [115]. Therefore, interconnections between ROS and retrograde pathways lead to the regulation of stress-responsive regulatory mechanisms. In contrast to retrograde signaling, ROS-mediated activation of the MPK4 cascade regulates downstream signaling to promote defense and acclimatization in stressful environments [116]. Overall, ROS triggers susceptibility and tolerance to stress at a specific level in plants by regulating retrograde and anterograde signaling.

ROS signaling at the organelle level is regulated by communication between (i) organelles and (ii) the organelle and the nucleus. ROS cannot travel long distances, but for communication purposes, mainly the following three mechanisms are involved: (i) organelles located in close proximity; (ii) connections between organelles and the nucleus through tube-like extensions; and (iii) communication between organelles through protein complexes and in the presence of aquaporins [35].

#### 4.2.1. Chloroplast: The Major Site of ROS Production

**Figure 5:**
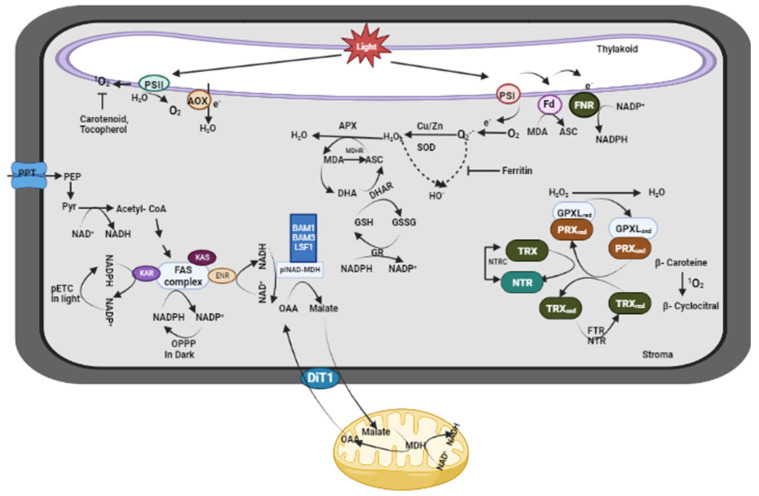
ROS regulation in the thylakoid and stroma regions of the chloroplast. It illustrates ROS production sources, including photosynthetic electron transport and photoreduction processes in the thylakoid membranes, and highlights ROS scavenging mechanisms such as ascorbate peroxidase and glutathione reductase in both the thylakoid lumen and the stroma. Pyr: pyruvate; PPT: phosphoenolpyruvate/phosphate translocator; BAM: Beta-Amylase; DiT1: dicarboxylate transporter 1; ENR: enoyl-acyl carrier protein (ACP) reductase; FAS: fatty acid synthase; OPPP: oxidative pentose phosphate pathway; PEP: phosphoenolpyruvate; KAR: β-ketoacyl-ACP reductase; KAS: β-ketoacyl-ACP synthase; OAA: oxaloacetate; plNAD-MDH: plastidial NAD-dependent malate dehydrogenase; PSI, II: Photosystem I, II; pETC: Plastid electron transport chain; MDHR: monodehydroascorbate reductase; DHA: Docosahexaenoic acid; GSH: Glutathione; GSSG: Glutathione disulfide; FNR: Ferredoxin-NADP+-reductase; Fd: Ferredoxin; NTR: Nitroreductases; TRX: Thioredoxin; PRX: Peroxiredoxin; SOD: Superoxide dismutase.

The chloroplast is considered the major site of ROS production. Energy transfer from the excited state of P680 of photosystem II (PSII) to O_2_ leads to the production of singlet oxygen (^1^O_2_), which is scavenged by non-enzymatic antioxidants, carotenoids, and tocopherols [117,118]. The singlet state of oxygen leads to the breakdown of beta-carotene and direct retrograde signaling [119]. Photosystem I (PSI), as a donor, transfers one electron to oxygen and produces O_2_^•−^ in the presence of SOD, which on a thylakoid membrane is converted to H_2_O_2_ [120]. At the same time, membrane-bound thylakoid ascorbate peroxidase (tylAPX) detoxifies H_2_O_2_ to water, which is denoted as a water-to-water cycle [19]. In a study conducted by Dietz (2011), it was found that when adding two Cys peroxiredoxins to tylAPX, it is reduced to H_2_O_2_ (Figure 5). This peroxiredoxin is regenerated by TRX, GRX, and NTRC, which contain both NTR (NADPH-dependent thioredoxin reductase) and thioredoxin activity [121]. Further, TRX is reduced by NTR and the ferredoxin-dependent thioredoxin reductase (FTR) complex (Schurmann and Buchanan 2008). In the stroma, H_2_O_2_ is scavenged by antioxidants, APX, glutathione peroxidase-like (GPXL), and peroxiredoxin [120]. Recent research has highlighted the role of the chloroplast in mitochondrial ROS production. The *mosaic death1* (MOD1) gene in the chloroplast is involved in mitochondrial ROS production. The *MOD1* gene in the chloroplast encodes for the enoyl-acyl carrier protein (ACP) reductase in the fatty acid synthase (FAS) complex (Figure 5). The *mod1* mutant in *Arabidopsis* exhibited chlorotic curly leaves, dwarfism, early senescence, etc. under high temperatures [122]. To elaborate on the relationship between the chloroplast and mitochondria in *mod1* mutants, the *suppressor of mod1* (*som1*) was studied. In this study, three components were identified in the malate shuttle as a medium of interaction between both organelles, namely plNAD-MDH, DiT1, and mNAD-MDH1 (plastidial NADP-dependent malate dehydrogenase, chloroplast-dicarboxylate transporter 1, and mitochondrial NAD-dependent malate dehydrogenase 1). Therefore, in the *mod1* mutant, plNAD-MDH oxidizes accumulated NADH in the chloroplast to synthesize malate, and furthermore, there is an exchange between malate and OAA between the chloroplast and the mitochondria. In the mitochondria, malate produces NADH in the presence of mNAD-MDH1. NADH acts as a substrate for the ETC complex 1 and induces ROS production to control plant growth and, in extreme conditions, also leads to plant cell death (PCD).

#### 4.2.2. Mitochondria: ROS Production and Regulation

**Figure 6:**
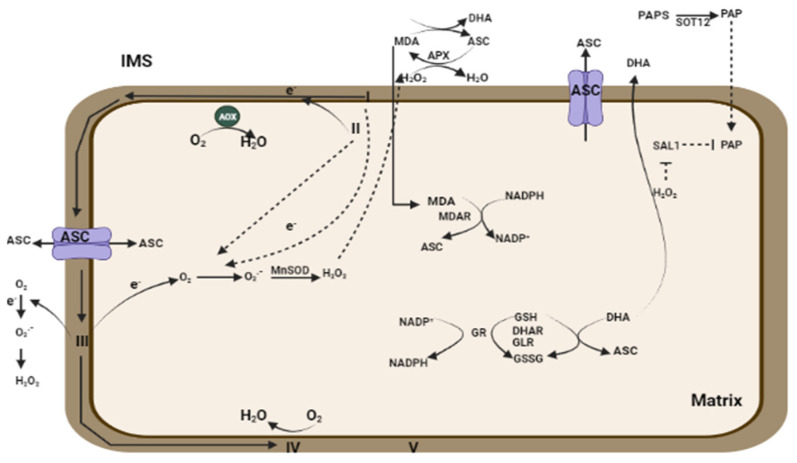
Regulation of ROS at the mitochondrial level. This comprehensive view emphasizes how ROS production and scavenging are balanced within mitochondria to maintain cellular health and prevent oxidative damage. Here, mETC regulates the production of ROS, which is scavenged in the presence of antioxidants. PAP: 3′-phosphoadenosine 5′-phosphate; MC9: METACASPASE 9; SOT12: SULFOTRANSFERASE 12; IMS: inner mitochondrial space; GLR: Glutamate receptor-like channels.

In the mitochondria, ROS production is firmly associated with the mETC, as complexes I, II, and III are majorly involved in O_2_^•−^ production [79] towards the matrix side, whereas complex III also produces O_2_^•−^ in the intermembrane space [123]. The last step of ascorbate (ASC) biosynthesis is completed in the inner mitochondrial space. Therefore, reduced ASC directly scavenges O_2_^•−^, which results in an alteration in the ASC redox condition [58]. In the presence of MnSOD, O_2_^•−^ is converted into H_2_O_2_, which is further processed by PRX and APX (Figure 6) [124,125]. To avoid mROS accumulation, there is a key step to bypass through e^-^ flow from complexes III and IV [79]. In the event of bypassing the e^-^ flow from complex III, the AOX role is very crucial [126]. Under stress conditions, AOX activation takes place by mETC in response to alleviated ROS derived from complex III [127,128]. An increase in AOX leads to an alteration in the redox state. The process also involves ANAC013 and ANAC017, located in the ER, being transported to the nucleus under stress conditions (Figure 6) [127,128]. An *aox1* mutant was reported as sensitive to drought and light stress in *Arabidopsis* with differentially expressed antioxidant genes in both the chloroplast and mitochondria [129].

#### 4.2.3. Peroxisomes: ROS Production and Scavenging Mechanisms

**Figure 7:**
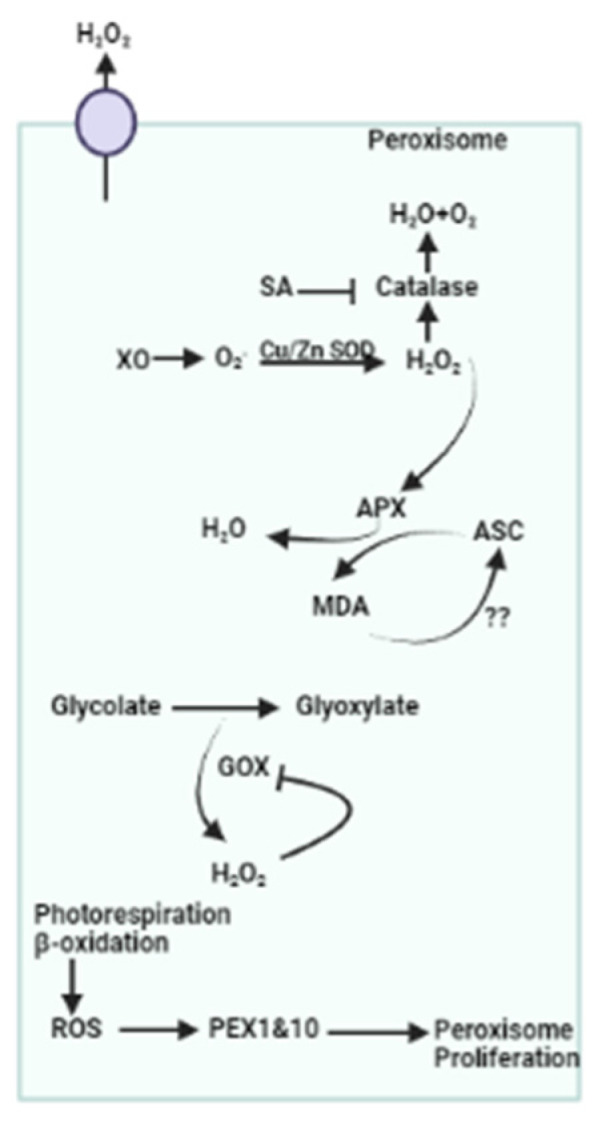
Role of peroxisomes in scavenging ROS. This figure highlights the involvement of peroxisomal antioxidant systems and their interaction with other cellular antioxidant networks. XO: xanthine oxidase; SA: salicylic acid; PEX: peroxin; APX: ascorbate peroxidase, MDA: Malondialdehyde; ASC: Ascorbate; GOX: Glycolate oxidase; ROS: Reactive oxygen species.

In the peroxisome, the accumulation of ROS takes place via several pathways, which are maintained by scavenging mechanisms. During water scarcity, the CO_2_ and O_2_ ratios become altered in mesophyll cells, and photorespiration also increases. Glycolate, synthesized in the chloroplast, is transported to the peroxisome for its oxidation, which results in H_2_O_2_ accumulation (Figure 7) [130,131]. Apart from this, beta-oxidation, SOD, and flavin oxidase induce the production of H_2_O_2_ in the peroxisome [132]. Catalase is a key antioxidant enzyme in the peroxisome that regulates H_2_O_2_ levels. Although H_2_O_2_ scavenging is also regulated by APX and the asada cycle, in this study, a poor contribution was reported as the *apx3* mutant reported no phenotypic changes in *Arabidopsis* [133,134]. In another study, the role of polyamine oxidases was also reported to balance ROS production and scavenging under drought stress, as altered expression of drought-responsive genes was identified in peroxisomal *pox*-mutated plants (Figure 7) [135].

#### 4.2.4. Vacuole: Role in ROS Regulation and Redox Homeostasis

The vacuole is considered a sink of H_2_O_2_, which is balanced by type III peroxidase activity [58]. Here, phenoxyl radicals are reduced in the presence of ascorbate and produce DHA, which is transported to the cytosol [136]. Therefore, we can conclude that the vacuole could maintain redox homeostasis under stress conditions to improve the tolerance capability of plants.

## 5. How Do Reactive Oxygen Species (ROS) Cause Oxidative Damage to Macromolecules?

ROS accumulation under stressful situations leads to oxidative stress, resulting in oxidative impairment of macromolecules like DNA, proteins, lipids, etc. Damage to these macromolecules can cause alteration in membrane fluidity, base substitution, structural and functional changes in proteins, etc., followed by plant cell death [137].

Under stress, lipid peroxidation accelerates oxidative damage as a result of enhanced ROS accumulation. After the peroxidation of polyunsaturated fatty acids (PUFA), malondialdehyde induces membrane damage [138]. In the phospholipid, an ester bond between glycerol and fatty acid and a double bond between C-C are the two common sites for ROS to attack. Lipid peroxidation is a three-step process that consists of an initiation, progression, and termination phase [139]. Firstly, a reaction between ^●^OH radicals, derived from O_2_, and PUFA produces PUFA alkyl radicals, which convert into peroxyl radicals in the presence of molecular oxygen. Further, PUFA peroxyl radicals interact with PUFA-H and generate lipid hydroperoxide. Lipid hydroperoxide then reacts with reduced metals, such as Fe^+2^, undergoes reductive cleavage, and produces lipid alkoxy radicals, lipid epoxide, alcohol, alkene, aldehydes, etc. In the last step of lipid peroxidation, lipid-derived radicals produce fatty acid dimers and peroxide bridge dimers [140,141,142,143], which led to disruptions in the permeability and fluidity of the membrane.

Under excessive ROS conditions, the oxidation of proteins can be divided into four steps, namely (i) oxidation catalyzed by metal, (ii) oxidation of amino acids, (iii) cleavage induced by oxidation, and (iv) forming a conjugate with a product of lipid peroxidation. To generate H_2_O_2_, reduction and oxidation of metal ions Cu(II)/Cu(I) and Fe(III)/Fe(II) are catalyzed by NAD(P)H oxidase. Furthermore, the oxidized form of metal ions interacts with proteins at their metal binding site and generates ^●^OH radicals in the presence of H_2_O_2_, which results in peptide bond breakage [143]. In addition, ROS mediates the oxidative PTM of the thiol group of cysteine and methionine, which play a crucial role in ROS sensing, as previously discussed. Conjugation with lipid peroxidation products like MDA and 4-hydroxynonenal (HNE) can also modify proteins indirectly [144]. Tissue injury due to oxidative stress also induces a concentration of carbonylated proteins, which is widely used to indicate the extent of oxidation of proteins [145].

DNA base and sugar moieties are the most susceptible targets of ROS under stress conditions. The ^●^OH radicals interact with DNA bases and the deoxyribose backbone [138] and generate mainly 8-hyroxyguanine. Oxidative damage to DBA also induces mutations by inducing specific alterations at G:C sites. Apart from this, lipid peroxidation also targets DNA bases because of indirect ROS attacks [146]. Another target of ROS is DNA sugar, where ROS attacks the C4 position of deoxyribose sugar and releases hydrogen atoms to produce deoxyribose radicals, which results in a break in the DNA strand [147]. In in vivo experiments, ROS toxicity is mainly the result of the Fenton reaction, where ^●^OH radicals produce and attack DNA or its associated proteins, which cannot be repaired easily. As compared to nuclear DNA, chloroplast and mitochondrial DNA are more prone to damage by oxidation due to a lack of histones and other protective proteins [148]. Excessive oxidative damage induced by ROS can damage DNA permanently, even in the presence of a repair system, which can affect cells negatively.

## 6. ROS Signaling Dynamics: Intracellular and Intercellular Communication

Reactive oxygen species are scavenged rapidly at the cellular level, which means their diffusion is not possible over longer distances. Transmission of ROS can be mediated as an ‘altered ROS state’ within or between the cell(s) or along the membrane. The coupling of production, sensing, and transportation is termed a “ROS wave”, which is different from the term “diffusion”. Here, diffusion is regarded as the mobilization between different locations. In contrast, the ROS wave is in an active antipropagation state along with tissues and across cells, which can include different signaling networks [149,150,151,152,153,154,155]. In an additional study, improved acclimatization was reported through the integration of signals induced by ROS waves under two different stresses, originating in different tissues of *Arabidopsis* plants [154]. This study concluded the integration of ROS signals through intracellular networks and their coordination in the physiological responses of plants [96,97].

## 7. Reactive Oxygen Species (ROS) in Yeast: Insights into Eukaryotic Biology

Electron leakage from the mETC leads to the source of ROS accumulation. In yeast, there is the absence of complex I, but in the matrix region of mitochondria, there are three NADH dehydrogenases that are insensitive to rotenone [156]. The NADH in the matrix region is oxidized in the presence of inner NADH dehydrogenase (NDI1). The Ndi1P regulates redox balance by maintaining the level of NADPH in mitochondria, produced by the TCA cycle [157]. Two external NADH dehydrogenases (Nde1/2) oxidize NADH in the cytosol, produced by glycolysis, similar to plant mitochondria [158]. Glycolysis derived by NADH in the cytosol can also be oxidized by the glycerol-3 phosphate dehydrogenase (G-3PDH) shuttle [159]. NADH dehydrogenase, involved in respiration, generates superoxide anions. These anions are converted into H_2_O_2_ in the presence of SOD2P and SOD1P in the matrix and cytosol, respectively [160]. The apoptosis-inducing factor (Aif1P) in yeast, homologous to AIF1 in mammals, may have antioxidant properties in mitochondria as it has an oxidoreductase domain similar to AIF1 [160]. Reduced cerebellar granule cell death was reported in overexpressed AIF1 cells under peroxide stress, which indicates its ROS scavenging property [161]. On the contrary, after treatment with H_2_O_2_ (0.4 mM), massive cell death was reported in overexpressed Aif1P yeast cells [162], which also indicates the probability that AiF1P does not reduce ROS, at least in the H_2_O_2_ form [162]. To verify the ROS defense mechanism of AiF1P, analysis needs to be performed by exposing different types and concentrations of ROS to Aif1P overexpressed cell lines.

## 8. Yeast Responses to Oxidative Stress: Regulatory Mechanisms

In yeast, H_2_O_2_ regulation chiefly belongs to thiol peroxidases, which are comprised of PRX and GPXL as non-heme antioxidant enzyme subgroups [163,164]. Mainly, two TFs are involved in regulating redox homeostasis, i.e., Yap1 in *Saccharomyces cerevisiae* (Pap1 in *Schizosaccharomyces pombe*) and SKN7. Msn2/4 TFs are also involved in the regulation of oxidative stress-related receptors [165]. Regarding ROS accumulation, activation of Skn7 depends on the phosphorelay system [166,167]. Activated Skn7 binds to dynamin-related GTPases (DNM1) and OLA1 (ATPase) promoters and regulates stress-responsive genes [168]. Simultaneously, Skn7 and Yap1 activate genes like TRXL, GPXL, CTT1, etc. to detoxify oxidative stress [168,169]. The DNA-binding domain of Skn7 is homologous to Hsf1. Therefore, their interaction activates the regulation of HSPs under oxidative stress [170]. Under hyperosmolarity, there is a reduction in the kinase activity of Sln1, which promotes phosphorylation in yeast, and dephosphorylated Sln1 accumulates. The phosphoryl group becomes transferred from histidine to aspartate and then to histidine 64 of Ypd1, a phosphotransferase [167,171,172]. Phosphorylated Ypd1 then phosphorylates Ssk1 and Skn7 response regulators. After phosphorylation, Skn7 regulates the expression of TRX2 [166]. Another key TF, Yap1, a bZip TF, plays a key role in regulating oxidative stress. Yap1 and orp1 (oxidant receptor per oxidant1 or GPX3) regulate redox status [173]. In the presence of H_2_O_2_, the thiol group (-SH) of cys is replaced by -SOH in orp1, which further forms an intramolecular disulfide bond in Yap1. There is a conformational change in Yap1, which is not recognized by crm1 (the nuclear export signal) and regulates the accumulation of modified Yap1 in the nucleus [174]. Here, ybp1 binds to Yap1 and regulates its oxidation by orp1 [175,176]. Yap1 in its oxidized form binds to the Yap1 response element (YRE) in the nucleus and regulates Yap1 regulon expression. Further, it is regulated by TRX1/2 and is released from the nucleus to the cytoplasm [177]. In *S. pombe*, oxidative stress is regulated by, firstly, PAP1, homologous to Yap1, and secondly, Sty1, a protein kinase. Pap1 acts as an early regulator but reduces its activity as H_2_O_2_ concentrations rise. It is oxidized by the PRX-TRX1 sensor under oxidative stress [178,179]. Under high H_2_O_2_ concentrations, the thiol group on cys in TRX converts to sulphinic acid, becomes inactivated, and reduces Pap1 activity [180]. In the presence of Sty1-regulated Srx1, the relay system becomes reactivated via the reduction in the sulphinic group of TRX [180]. Under severe oxidative stress, the Sty1-wis1-wak1/wis4/1 MAPK cascade regulates stress regulatory genes via phosphorylation of different TFs and regulators [181]. Prr1, an Skn7 homolog, regulates oxidative stress in fission yeast and the activation of Pap1 or Atf1-dependent genes [182,183].

## 9. The Upside of Reactive Oxygen Species: Key Benefits

**Figure 8:**
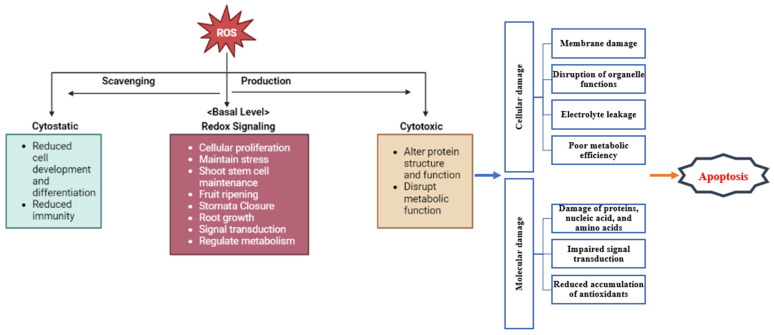
Role of ROS in the regulation of plant growth and development. Here, it is exhibited that ROS supports plant development positively at its basal level. ROS concentrations below and above the basal level can disrupt plant mechanisms at cellular and physiological levels.

Under normal conditions, ROS is also essential to controlling plant growth, development, and different cellular responses [184,185,186]. At below and above threshold levels, ROS deteriorates cellular growth and proliferation. Maintenance of the threshold or basal level is indeed an intermediate state between cytostatic and cytotoxicity [18]. The basal-level limitation of ROS signals may be the reason behind the evolutionary development of ROS signaling in the apoplast (Figure 8). Activation of RBOH and aggregation of ROS in the apoplast as a result of a ROS wave prevent diffusion of ROS across the plasma membrane [187,188,189]. The subgroup of RBOH/NOX has evolved to produce ROS at the external side of the plasma membrane in the apoplast region, away from the nucleus [190,191,192,193]. In the aerobic organism, ROS were identified as sensors to detect oxygen levels, which comply with the importance of ROS in the direction of cellular processes like proliferation, growth, development, aging, and differentiation [194,195,196,197] (Figure 8).

## 10. *Poaceae* Family and Drought Stress: ROS Mitigation

*Poaceae*, which is commonly known as grasses, is a large family of monocotyledons and includes crops like sugarcane and cereals such as wheat, rice, barley, and maize. All these crops are quite susceptible to abiotic stress [198]. Global warming is one of the key responsible factors for the drastic environmental changes imposing stress on plants, among which scarcity of water is the most concerning factor in the growth and development of plants, which leads to oxidative stress [199]. Among the numerous adaptive mechanisms to mitigate stress conditions, ROS regulation is one of the key strategies. To improve stress adaptation or enhance tolerance, genetic engineering has provided a step beyond the traditional breeding system, i.e., the generation of transgenic or gene-edited lines. Genetic engineering provides an understanding of stress regulation in a gene-responsive manner, which can be further manipulated in transgenic crops to improve tolerance. Through decades of research, several stress-responsive genes have been studied in *Poaceae* plants that mitigate drought stress by regulating ROS homeostasis by improving enzymatic or non-enzymatic antioxidant scavenging mechanisms in the plants (Table 2).

*Deschampsia antarctica* is a colonized *Poaceae* grass in Antarctica. Overexpression of galactinol synthase, *DaGolS2* and *OsGolS2*, in rice resulted in enhanced tolerance to drought and cold by reducing ROS levels compared to the wild-type plants [200]. Cytokinins are one of the phytohormones that regulate plant growth and development. Partial silencing of *ShCKX*, cytokinin dehydrogenase, resulted in enhanced antioxidant levels in transformed sugarcane under water-deficient conditions [201]. Ferritins have been reported to be present in all types of cells [222], as ferritin genes are conserved throughout the plant kingdom [223]. Under heat, drought, and oxidative stress, *TaFER-5B* transcription was induced [202]. Furthermore, overexpression of *TaFER-5B* in wheat plants has been reported to enhance tolerance under heat, drought, iron, and oxidative stress [202]. Similarly, overexpression of *AtBBX29* in sugarcane reported an improved antioxidant defense mechanism to scavenge drought-induced ROS accumulation [203]. MYB transcription factors regulate plant growth and development and even environmental stress conditions [224]. Overexpression of *OsMYB55* in maize resulted in improved ROS scavenging under drought and heat stress [204]. Under drought and salt stress, *SRG7*, a stress-related gene, was identified to be significantly upregulated. Its overexpression in maize reported enhanced drought and salt tolerance with increased antioxidant content in transgenic lines [218].

Using CRISPR/Cas9, the loss of function of SAPK2, an ABA-activated protein kinase, and SAP, a senescence-associated protein, was performed in rice [205,206], which resulted in mutated lines that are more sensitive to drought due to ROS accumulation than the wild-type plants. Under severe abiotic stress, the highly upregulated activity of superoxide dismutase (SOD) was observed in mangroves. Transgenic lines of rice were generated by expressing *AmSOD1* and resulted in better drought and salt tolerance [207]. The target of stress-responsive NAC2 (SNAC2), ornithine-delta-aminotransferase (delta-OAT), was overexpressed in rice, which resulted in tolerance with regard to drought and oxidative stress [208]. Like the previous study, *OsSRO1G*, similar to RCD1 in rice and a target gene of SNAC1, was overexpressed in rice and resulted in improved tolerance to drought and oxidative stress by regulating the closure of stomata and the accumulation of H_2_O_2_ [24]. Overexpression of the *phospholipase D alpha1-E2* gene in rice protected the transgenic plants when exposed to water limitations through the prevention of ROS accumulation [209]. Similarly, overexpression of *OsMT1-alpha* (metallothioneins), *OsHAK1* (high-affinity potassium transporter), and *OsPUB67* (U-box E3 ubiquitin ligase) enhanced drought tolerance and ROS scavenging ability in transgenic lines compared with wild-type plants [28,210].

In another study, the gene coding for the abscisic stress ripening (ASR) protein, which regulates abiotic stress tolerance in plants, was overexpressed in wheat. Overexpression of *TaASR1-D* resulted in transgenic lines with improved drought, salt, and osmotic tolerance through reinforced antioxidant capacity [221]. The *OsDRZ1*, *drought-responsive zinc finger protein 1* gene, is a translational repressor. Overexpression of *OsDRZ1* resulted in increased drought tolerance and reduced ROS accumulation in transgenic lines. On the contrary, the silencing of *OsDRZ1* by RNA interference reduced antioxidant activities and enhanced drought sensitivity [212]. Overexpression of some transcription factors is also identified as drought stress regulators. Overexpression of *OsbZIP62* and *SbNAC2* in rice confirmed their role as key stress response regulators by improving drought tolerance and inducing antioxidant enzyme activity in transgenic lines [213,220]. Like the above study, overexpression of *SbNAC9* in sorghum reported improved drought tolerance, root system, and antioxidant enzyme activities in transgenic lines compared with the wild-type [219]. EBP89, an ethylene-responsive element-binding protein, is a member of the AP2/ERF subfamily. The *OsERP89* gene is reportedly involved in the regulation of drought stress, as was confirmed when *OsEBP89* was knocked out in rice, which improved ROS scavenging properties and drought tolerance in the transgenic lines [214]. Additional studies targeted ferrochelatase (FC1/2), a terminal enzyme for the biosynthesis of heme, where free heme acts as an important chloroplast for nuclear signals. Overexpression of *HvFC1* and *HvFC2* in barley demonstrated their role in enhancing drought stress tolerance through the overexpression of genes coding for antioxidants that detoxify ROS [216]. In salicylic acid biosynthesis, isochorismate synthase (ICS) plays an important role in mediating stress responses in plants. Overexpression of *ICS* in barley resulted in enhanced drought tolerance and ROS mitigation, whereas increased levels of ABA were reported in wild-type and *ICS*-silenced barley plants with poor water retention when exposed to drought stress [217]. Similarly, overexpression of *HvGST4*, a glutathione-s-transferase, in barley resulted in improved abiotic stress tolerance by improving ROS scavenging [215].

## 11. Conclusions and Emerging Trends

**Figure 9:**
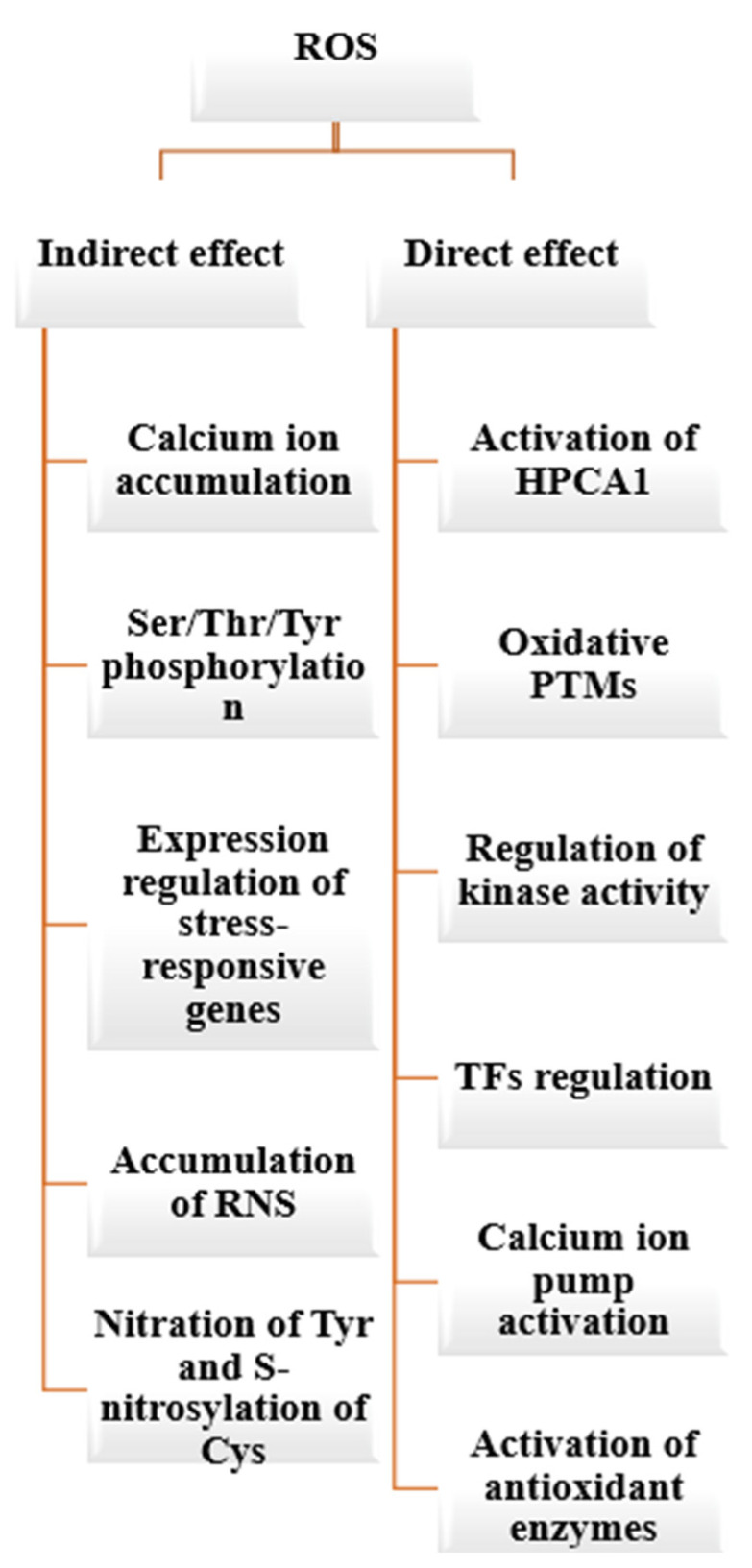
Indirect and direct effects of ROS on plant regulatory mechanisms. It shows how ROS influence cellular processes through direct interactions with biomolecules, such as proteins, lipids, and nucleic acids, leading to changes in their function and stability. Additionally, this figure highlights the indirect effects of ROS through the modulation of signaling pathways and gene expression, impacting plant responses to stress and development.

Excessive oxidative stress hampers plant fitness due to the development of ROS, which directly or indirectly affects the regulatory mechanisms of plants (Figure 9). ROS accumulate under abiotic stress conditions, causing oxidative damage and eventually cell death. ROS are also key players in the complex signaling network of plants’ stress responses. They are involved in signal transduction, where finely tuned regulation networks maintain ROS at non-toxic levels in a balancing act between ROS production through ROS-generating enzymes and the required production of ROS during basic cellular metabolism and ROS-scavenging pathways.

In the upcoming decades, the challenge will be to develop crops that can be sustainably cultivated under ever-changing, harsh environmental conditions. Molecular and physiological responses in crop plants in response to abiotic stresses need to lead to adaptation to counter oxidative conditions or the toxic effects of ROS. What strategic action we should take to improve crop productivity under abiotic stress conditions and nullify ROS toxicity is still unclear, and no one solution exists. Targeting and enhancing the antioxidant defense is one strategy to enhance crop stress tolerance and yield. Several examples where genes coding for enzymatic antioxidants, osmolytes, and transcription factors were overexpressed in *Poaceae* crops, resulting in enhanced drought tolerance, have been reported and should be explored further. The review has summarized all the possible mechanisms regulated through ROS, both directly and indirectly, to regulate stress conditions. Here, the ROS–ABA-Ca^+2^ relationship has also been extensively explained. This review can mediate the development of strategies to modify crops to counteract the toxic effects of ROS accumulation under abiotic stress conditions, which could include new cultivation methods, arranging diverse cropping welfare schemes, and diverse non-customary and customary paths. In attempts to acquire climate-resilient crops able to withstand frequent and extreme heat and drought conditions, researchers are currently focused on genome-wide association strategies, genome-wide selection with great throughput-based phenotype and genotype strategies, and gene editing, which will be crucial to increasing our understanding and identifying genes that can potentially play a role in reducing oxidative stress damage and stress adaptation in crops.

## Figures and Tables

**Table 1:** Enzymatic and non-enzymatic antioxidants with ROS scavenging activity.

Antioxidants	Catalytic Reaction Sites in Different Subcellular Localization	Activity	References
Enzymatic			
Superoxide dismutase (SOD) {EC 1.15.1.1}	Peroxisome, Mitochondria, Cytosol, and Chloroplast	Disproportionate hydrogen peroxide from the superoxide anion	[77]
Catalase {EC 1.11.1.6}	Peroxisome and Mitochondria	Scavenges of H_2_O_2_	[78]
Ascorbate peroxidase (APX) {EC 1.11.1.11}	Peroxisome, Mitochondria, Cytosol, and Chloroplast	Catalyze H_2_O_2_ into O_2_ using ascorbate as an electron-specific donor	[79,80]
Monodehydroascorbate peroxidase (MDHAR) {EC 1.6.5.4}	Chloroplast, Mitochondria, and Cytoplasm	Regenerates AsA by catalyzing the reduction in monodehydroascorbate using NADH or NADPH as an electron donor	[81]
Dehydroascorbate reductase (DHAR) {EC 1.8.5.1}	Mitochondria, Cytoplasm, and Chloroplast	Regenerating ASC from an oxidized state	[82]
Glutathione reductase (GR) {EC 1.6.4.2}	Cytoplasm, Mitochondria, and Chloroplast	Catalyzes the reduction in GSSG and GSH using NADPH as a reducing co-factor	[83]
Glutathione peroxidase (GPX) {EC 1.11.1.7}	Chloroplast, ER, Mitochondria, and Cytoplasm	Reduces H_2_O_2_ to water	[82]
Non-enzymatic antioxidants			
Ascorbic acid (AA)	Mitochondria, Chloroplast, Cytosol, Apoplast, and Vacuole	Detoxify H_2_O_2_ via the action of APX	[84]
Reduced glutathione	Mitochondria, Chloroplast, Cytosol, Peroxisome, Vacuole, and Apoplast	Serve as a detoxifying co-substrate for enzymes like PX, GR, and GST	[82]
Alpha-Tocopherol	Membrane	Detoxifies products of membrane lipid peroxidation	[85,86]
Flavonoids	Vacuole	Scavengers of H_2_O_2_, ^1^O_2_, and OH^●^	[87,88]
Carotenoids	Chloroplast	Quenches excess energy from photosystems and light-harvesting complexes.	[89]
Proline	Cytosol, Chloroplast, Mitochondria, and Plasma Membrane	Scavenger of ROS to prevent damage due to lipid peroxidation	[90]
Plastoquinone/Ubiquinone	Thylakoids of Chloroplasts and the inner membrane of Mitochondria	Transport of electrons in ETC flux during photosynthesis and aerobic respiratory chain	[80,91]

**Table 2:** Genetically engineered *Poaceae* crops to enhance drought tolerance by manipulating transgenes linked to the control of ROS toxicity during the stress response. The listed “Outcome” refers to the response of the stressed plants in terms of ROS accumulation and antioxidant scavenging activity that contribute to the overall tolerance or susceptibility.

Crop	Gene	Construct	Outcome	Stress	Reference
Rice	*DaGolS2*, *OsGolS2*	Overexpression	Tolerant: ↓ H_2_O_2_; ↑ *SODcc2* + *CatB* expression; ↑ RFO	Drought, salt	[200]
Sugarcane	*ShCKX*	Partial silencing	Tolerant: ↓ H_2_O_2_; ↑ SOD + CAT + Proline	Drought	[201]
Wheat	*TaFER-5B*	Overexpression	Tolerant	Drought, Heat	[202]
Sugarcane	*AtBBX29*	Overexpression	Tolerant: ↓ H_2_O_2_; ↑ SOD + CAT + Proline	Drought	[203]
Maize	*OsMYB55*	Overexpression	Tolerance	Drought/Heat	[204]
Rice	*Ossapk2*	Loss of function	Sensitive: ↑H_2_O_2_ + O_2_^−^; ↓ SOD + CAT + POD +APX	Drought	[205]
Rice	*OsSAP*	Loss of function	Sensitive: ↑H_2_O_2_ + O_2_^−^; ↓ SOD + CAT + POD + Proline	Drought	[206]
Rice	*Cu/Zn SOD*	Overexpression	Tolerant: ↑ Survival + SOD isozyme	Drought, salt	[207]
Rice	*OsSRO1c*	Overexpression	Tolerant: ↑ Stomatal closure; ↑ H_2_O_2_ in guard cells; ↓ *DST* expression	Drought	[24]
Rice	*OsOAT*	Overexpression	Tolerant: ↑ POD + GSH + GPX + chlorophyll; ~ SOD + CAT	Drought	[208]
Rice	*OsPLDα1*	Overexpression	Tolerant: ↑ Stomatal closure; ↑ *SOD* expression	Drought	[209]
Rice	*OsPUB67*	Overexpression	Tolerant: ↑ Proline + SOD + POD; ~ CAT; ↑ Stomatal closure	Drought	[28]
Rice	*OsHAK1*	Overexpression	Tolerant: ↓ H_2_O_2_; ↑ POX + CAT + Proline; ↑ *Pox1* + *CatA* + *CatB* and TF (*SNac2* + *Zip23* +*Myb2* + *Dreb2A*) expression	Drought	[210]
Rice	*OsMT1a*	Overexpression	Tolerant: ↑ APX + CAT + SOD	H_2_O_2_ exposure	[211]
Rice	*OsDRZ1*	Overexpression	Tolerant: ↓ H_2_O_2_ + O_2_^−^; ↑ POD + SOD + Proline + Stomatal closure; ↑ *Pod* + antioxidant-related gene expression	Drought	[212]
Rice	*OsbZIP62*	Overexpression	Tolerant: ↓ H_2_O_2_; ↑ *GL1 + NAC10* + *DSM2* expression; Interact with SAPKs	Drought	[213]
Rice	*OsEBP89*	Loss of function	Tolerance: ↓ H_2_O_2_; ↑ SOD + Proline; ↑ *Apx1* + *GPx* + *P5CS* + *HS TF* expression	Drought	[214]
Barley	*HvGST4*	Loss of function	Susceptible: ↑ H_2_O_2_ + O_2_^−^; ↓ GSH	Abiotic	[215]
Barley	*HvFC1/2*	Overexpression	Tolerant: ↑ *Sod* + *Cat* expression; ~ Stomatal closure	Drought	[216]
Barley	*HvICS*	Overexpression	Tolerant:↓ H_2_O_2_ + O_2_; ↑ SOD + CAT + APX + POD	Drought	[217]
Maize	*ZmSRG7*	Overexpression	Tolerant:↓ H_2_O_2_ + O_2_; ↑ SOD + POD; ↑ expression ABA pathway genes	Drought, salt	[218]
Sorghum	*SbNAC9*	Overexpression	Tolerant:↓ H_2_O_2_ + O_2_; ↑ SOD + POD	Drought	[219]
Rice	*SbNAC2*	Overexpression	Tolerant: ↑ SOD + POD + CAT; ↑ *Zip23 + DREB1/2A + Lea3* expression	Drought	[220]
Wheat	*TaASR1-D*	Overexpression	Tolerant:↓ H_2_O_2_ + O_2_; ↑ SOD + CAT + GPx; ~ POD; ↓ Proline	Abiotic	[221]

Note: ↑ Increase in concentration, ↓ Decrease in concentration, ~ No change in concentration.
